# Permanent pacemaker implantation in unexplained syncope patients with borderline sinus bradycardia and electrophysiology study‐proven sinus node disease

**DOI:** 10.1002/joa3.12460

**Published:** 2020-11-22

**Authors:** Ioannis Doundoulakis, Konstantinos A. Gatzoulis, Petros Arsenos, Polychronis Dilaveris, Ioannis Skiadas, Dimitrios Tsiachris, Christos‐Konstantinos Antoniou, Stergios Soulaidopoulos, George Karystinos, Voula Pylarinou, Maria Drakopoulou, Skevos Sideris, Charalambos Vlachopoulos, Dimitrios Tousoulis

**Affiliations:** ^1^ First Department of Cardiology National and Kapodistrian University “Hippokration” Hospital Athens Greece; ^2^ Athens Heart Center Athens Medical Center Athens Greece; ^3^ State Department of Cardiology “Hippokration” Hospital Athens Greece

**Keywords:** electrophysiology study, sinus bradycardia, sinus node dysfunction, syncope

## Abstract

**Background:**

Significant sinus bradycardia (SB) in the context of sinus node dysfunction (SND) has been associated with neurological symptoms. The objective was to evaluate the effect of permanent pacing on the incidence of syncope in patients with rather mild degrees of SB, unexplained syncope, and “positive” invasive electrophysiologic testing.

**Methods:**

This was an observational study based on a prospective registry of 122 consecutive mild SB patients (61.90 ± 18.28 years, 61.5% male, 57.88 ± 7.73 bpm) presenting with recurrent unexplained pre and syncope attacks admitted to our hospital for invasive electrophysiology study (EPS). Τhe implantation of a permanent antibradycardia pacemaker (ABP) was offered to all patients according to the results of the EPS. Eighty patients received the ABP, while 42 denied.

**Results:**

The mean of reported syncope episodes was 2.23 ± 1.29 (or presyncope 2.36 ± 1.20) in the last 12 months before they were referred for a combined EP guided diagnostic and therapeutic approach. Over a mean follow‐up of approximately 4 years (50.39 ± 32.40 months), the primary outcome event (syncope) occurred in 18 of 122 patients (14.8%), 6 of 80 (7.5%) in the ABP group as compared to 12 of 42 (28.6%) in the no pacemaker group (*P* = .002).

**Conclusions:**

Among patients with mild degree of SB and a history of unexplained syncope, a set of positivity criteria for the presence of EPS defined SND after differentiating reflex syncope, identifies a subset of patients who will benefit from permanent pacing.

## Graphical abstract

Among patients with mild degree of sinus bradycardia and a history of unexplained syncope, a set of positivity criteria for the presence of electrophysiology study defined sinus node dysfunction, identifies a subset of patients who will benefit from permanent pacing.

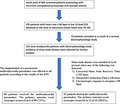

**Abbreviations**
1st AVB: 1st degree atrioventricular block
ABP: Anti bradycardia pacing
AVNCD: Atrioventricular node conduction defect
CSNRT: Corrected sinus node recovery time
EPS: Electrophysiology study
LBBB: Left bundle branch block
SACT: sinoatrial conduction time
SB: Sinus bradycardia
SND: Sinus node dysfunction

## 1 INTRODUCTION

Significant sinus bradycardia (SB) in the context of sinus node dysfunction (SND) has been associated with neurological symptoms such as dizziness, vertigo, or near syncope and syncope attacks.^1^, ^2^, ^3^ Syncope, defined as a transient loss of consciousness caused by transient global cerebral hypoperfusion, is a relatively common cause for seeking medical help, with visits progressively increasing with age.^4^

It has been suggested that a persistent fall in the heart rate of ≤50 Bpm or/and the presence of sinus pause of ≥3 seconds on a 12‐lead ECG and 24h Holter monitor recording respectively, during the workup of unexplained syncope, should be treated with permanent antibradycardia pacing (ABP).^4^ An ABP is also recommended when the electrophysiological study (EPS) reveals marked prolongation of the corrected sinus node recovery time.^4^ It is of note that official guideline recommendations for permanent pacing are not based on clinical studies. Other means of EPS derived evidence of SND such as sinoatrial conduction time (SACT) and the chronotropic response to atropine have not been included in the European and American guidelines for the management of unexplained syncope patient.^4^, ^5^, ^6^ Furthermore, we still lack clear answers to seemingly simple questions including the appropriate use of EPS and the exact criteria for pacing based on the results. In relation to the above, we have previously shown that a strong correlation exists between a variety of EPS derived SND parameters and even mild degrees of SB among such unexplained syncope patients.^7^ Whether these symptomatic patients with mild degrees of SB and EPS evidence of SND would benefit from ABP while avoiding the need for an implantable loop recorder to further establish the diagnosis, is a highly debatable issue.

The objective of the present “real‐world” study was to evaluate the effect of permanent pacing on the incidence of syncope in patients with mild degrees of SB, unexplained syncope and “positive” invasive electrophysiologic testing.

## 2 METHODS

**FIGURE 1:**
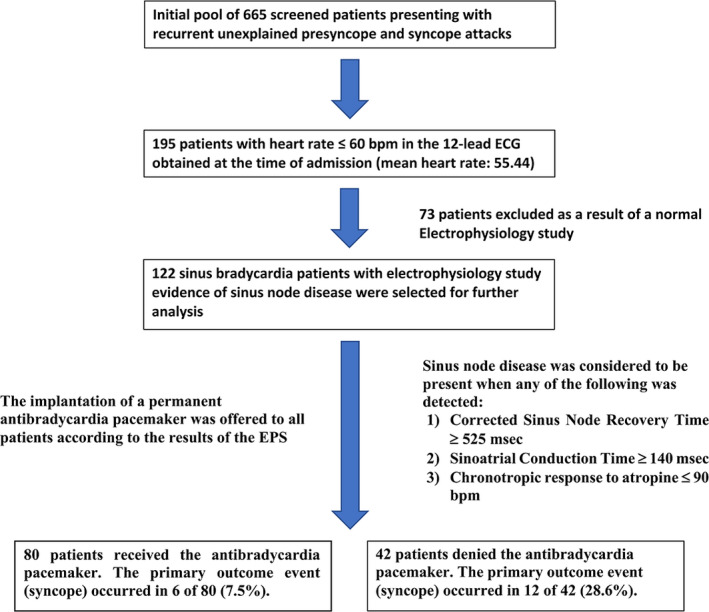
Flow chart of study and outcomes

This was an observational study based on a prospective registry of 665 screened patients (195 patients with heart rate ≤60 bpm in the 12‐lead ECG obtained at the time of admission, mean heart rate: 55.44), presenting with recurrent unexplained pre and syncope attacks admitted to our hospital for invasive EPS from 1995 until 2017 (Figure 1).

Among these 195 SB patients with a history of pre and syncope without a readily identifiable plausible cause (including overt sinus node disease or atrioventricular conduction disturbances evident on office or ambulatory electrocardiogram), a group of 122 SB patients with EPS evidence of SND (see below for the criteria of positivity) were selected for further analysis (73 patients excluded as a result of a normal EPS). Patients receiving bradycardia inducing agents such as B‐Blockers and/or Calcium channel blockers were excluded. The decision to implant a permanent pacemaker was made in all cases by the attending physicians. Patients with an indication for an implantable cardioverter defibrillator or cardiac resynchronization device were excluded. Protocol of the study was approved by our Research Ethical Committee and the informed written consents were taken from all patients after explaining the pros and cons of the EPS.

### 2.1 12‐lead electrocardiography and 24‐hour ambulatory monitor and EPS findings

The methodology of obtaining the noninvasive ECG features and the invasive EPS results has been previously published and has been consistently performed since the beginning of the study.^7^ The 12‐lead ECG resting heart rate was the one observed in the EP laboratory before the introduction of the EP catheters. The mean 24‐Hour ambulatory monitor derived heart rate was calculated after careful elimination of artifacts and any ventricular ectopic beats on appropriate template recognition patterns. Any sinus pauses <3 seconds were identified and noticed. SND was considered to be present when any of the following was detected:

1. Corrected Sinus Node Recovery Time (CSNRT) ≥525 msec
2. Sinoatrial Conduction Time (SACT) ≥140 msec
3. Chronotropic response to atropine ≤90 bpm

All patients underwent also atrioventricular node and His bundle function assessment in order to unmask the concurrent presence of atrioventricular node disease when any of the following abnormal values were obtained:

1. Βasic HV interval of ≥60 msec
2. Wenckebach cycle length ≥500 msec and 2:1 atrioventricular block cycle length ≥400 msec
3. Effective refractory period of the atrioventricular node ≥450 msec
4. Detection of infra‐Hisian block
5. Split His activity
6. Appearance of bifascicular or trifascicular block on atrial stimulation

The EPS protocol was completed with ventricular and supraventricular stimulation as well as carotid sinus massage according to the degree of clinical suspicion for an associated electrical disturbance. When the EPS derived diagnosis of SND was reached and in relation to other clinical laboratory features present such as patient age, clinical characteristics of the syncope episode suggesting an arrhythmic mechanism like the association with body injury in the absence of warning symptoms and or signs, a decision to proceed with ABP in the form of DDD and occasionally of AAI pacing was offered to the patient.

### 2.2 Patient follow‐up and outcome measures

Patients with ABP where followed up by at least 12‐month visits in the pacemaker clinic where telemetric interrogation of the device was performed regularly. In case of symptom recurrence and based on the degree of clinical suspicion, apart from the device interrogation to exclude malfunction, alternative causes of syncope recurrence were sought, occasionally repeating noninvasive testing such as tilt table testing or even EPS if complex ventricular arrhythmia in the device memory were detected. Any atrial high rate episodes detected by the device were noticed and recorded. Patients not receiving ABP were also followed up at least yearly with either clinic visits or/and telephone contacts. The study primary outcome measure was the time to the event of syncope (syncope‐free survival). Secondary outcome was time‐to‐death from any cause.

### 2.3 Statistical analysis

Continuous variables were summarized with mean and standard deviation (SD) and compared using independent samples *t* test or Mann‐Whitney *U* test as appropriate. Categorical variables were described with frequencies and percentage and compared using chi‐squared test. Kaplan‐Meier curves were used to visualize survival free from primary endpoint occurrence and the log rank test was applied to assess the presence of statistically significant differences. Multivariate Cox regression was used in order to assess and compare impact of parameters on survival free from primary endpoint occurrence. Significance level was set to *P* < .05 and two tailed. Data analysis was performed using the IBM/Statistical Package for Social Sciences (SPSS version 24; IBM, Chicago, IL, USA) program.

## 3 RESULTS

The clinical laboratory patients' characteristics are presented in Table 1. Most of the patients were elderly male without an underlying organic heart disease and with well‐maintained LVEF. The mean of reported syncope episodes was 2.23 ± 1.29 (or presyncope 2.36 ± 1.20) in the last 12 months before they were referred for a combined EP guided diagnostic and therapeutic approach. There were no statistical differences in the number of reported pre or/and syncope episodes in those who were later implanted with a pacemaker vs in those who did not receive a pacemaker. The 12‐lead ECG heart rate was 57.88 ± 7.73, and approximately 50% of patients had mean 24‐hour heart rate ≤60 bpm. A significant proportion of patients had associated conduction defects on the 12‐lead ECG in the form of 1st degree atrioventricular block (1st AVB), right or left bundle branch block (LBBB), bifascicular or trifascicular block. A 1st AVB, LBBB and bifascicular block were more frequently observed among pacemaker patients.

**TABLE 1:** Baseline patients' characteristics

Variables	Overall (N = 122)	No pacemaker (N = 42)	Pacemaker (N = 80)	*P*‐value
Age (years)	61.90 (±18.28)	55.98 (±21.97)	65.01 (±15.27)	0.070
LVEF (%)	57.36 (±9.81)	59.05 (±9.06)	56.46 (±10.13)	0.184
Sex (Male)	75 (61.50)	25 (59.50)	50 (62.50)	0.748
Presyncope (N of patients)	39 (32)	10 (23.80)	29 (36.30)	0.162
Syncope (N of patients)	92 (75.40)	34 (81)	58 (72.50)	0.303
Presyncope (N of events)	2.36 (±1.20)	2.30 (±0.95)	2.38 (±1.29)	0.937
Syncope (N of events)	2.23 (±1.29)	2.26 (±1.16)	2.21 (±1.36)	0.679
Follow‐up (months)	50.39 (±32.40)	44.74 (±25.73)	53.35 (±35.19)	0.400
Organic heart disease				
Coronary artery disease	26 (21.3)	7 (16.7)	19 (23.8)	0.364
Hypertrophic cardiomyopathy	1 (0.8)	0 (0)	1 (1.3)	0.467
Dilated cardiomyopathy	6 (4.9)	1 (2.4)	5 (6.3)	0.348
Valvular heart disease	3 (2.5)	0 (0)	3 (3.8)	0.204
12‐lead ECG				
Heart rate	57.88 (±7.73)	59.33 (±8.40)	57.11 (±7.29)	0.140
1st degree atrioventricular block	36 (29.5)	7 (16.7)	29 (36.3)	0.024
Right Bundle Branch Block	15 (12.3)	2 (4.8)	13 (16.3)	0.066
Left Bundle Branch Block	7 (5.7)	0 (0)	7 (8.8)	0.048
Left Anterior Hemiblock	17 (13.9)	3 (7.1)	14 (17.5)	0.117
Left Posterior Hemiblock	3 (2.5)	1 (2.4)	2 (2.5)	0.728
Bifascicular block	11 (9)	0 (0)	11 (13.8)	0.012
Trifascicular block	6 (4.9)	0 (0)	6 (7.5)	0.074
Holter monitoring				
Mean 24‐hour heart rate ≤60 bpm	59 (48.4)	21 (50)	38 (47.5)	0.793
Sinus pauses ≥2 sec	11 (9)	2 (4.8)	9 (11.3)	0.199
Second degree atrioventricular block	11(9)	3 (7.1)	8 (10)	0.436

The EPS results are presented in Table 2. Τhe implantation of a permanent ABP was offered to all patients according to the results of the EPS. Eighty patients received the ABP, while 42 denied. The two patient groups were overall well balanced, with no significant differences in EPS parameters. More than half of both patient groups had EPS evidence for an associated atrioventricular node conduction defect (AVNCD). Apart from 54 patients with CSNRT ≥ 525 msec, 28 patients had only SACT ≥ 140 msec and 29 patients had only chronotropic response to atropine ≤90 bpm, while 11 patients had both SACT ≥ 140 msec and chronotropic response to atropine ≤90 bpm.

**TABLE 2:** Results of the electrophysiological study

Variables	Overall (N = 122)	No pacemaker (N = 42)	Pacemaker (N = 80)	*P*‐value
CSNRT (msec)	503.63 ± 287.83	502.41 ± 356.56	504.26 ± 248.02	0.214
CSNRT ≥ 525 msec	54 (44.3)	15 (35.7)	39 (48.8)	0.168
SACT (msec)	182.50 ± 100.29	187.52 ± 138.47	178.88 ± 61.27	0.396
SACT ≥ 140 msec	50 (41)	18 (42.9)	32 (40)	0.760
Chronotropic response to atropine ≤90 bpm	52 (42.6)	19 (45.2)	33 (41.3)	0.672
Atrioventricular node disease	71 (58.2)	22 (52.4)	49 (61.3)	0.345

Abbreviations: CSNRT, Corrected sinus node recovery time; SACT, sinoatrial conduction time.

**FIGURE 2:**
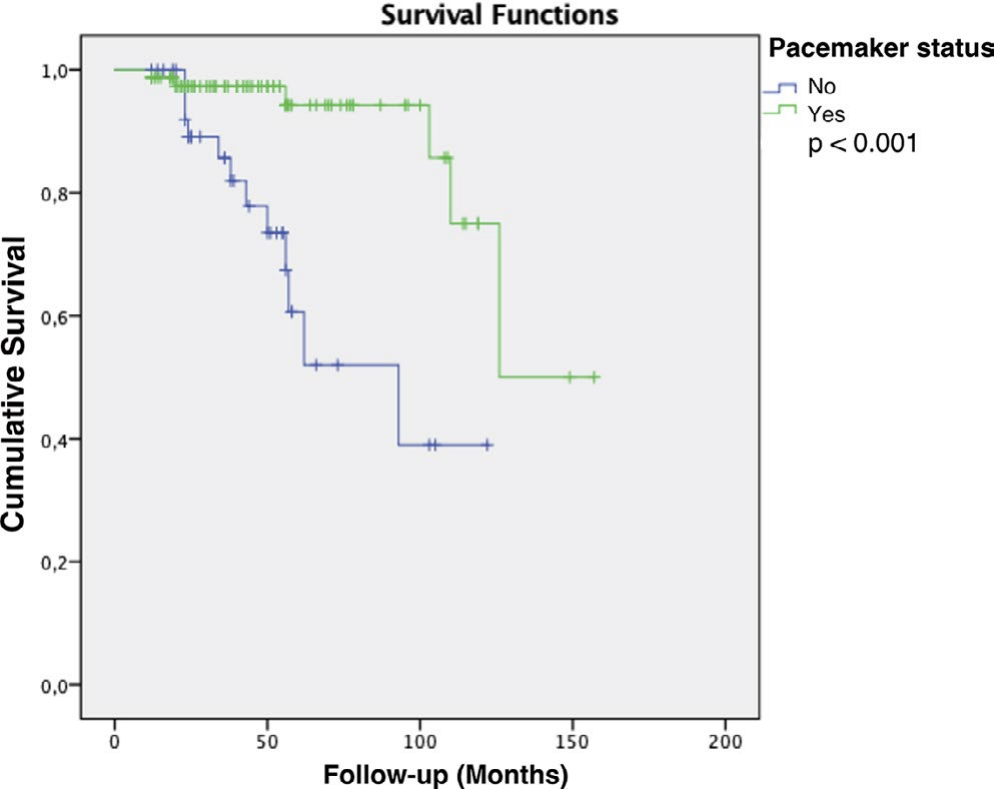
Kaplan‐Meier curves for syncope‐free survival according to permanent pacing status (primary endpoint)

Over a mean follow‐up of approximately 4 years (50.39 ± 32.40 months), the primary outcome event (syncope) occurred in 18 of 122 patients (14.8%), 6 of 80 (7.5%) in the pacemaker group as compared to 12 of 42 (28.6%) in the no pacemaker group (*P* = .002). Among 12 patients without pacing, 6 patients had only CSNRT ≥ 525 msec, 2 patients had only SACT ≥ 140 msec, and 1 patient had only chronotropic response to atropine ≤90 bpm, while 1 patient had both CSNRT ≥ 525 msec and SACT ≥ 140 msec, 1 patient had both CSNRT ≥ 525 msec and chronotropic response to atropine ≤90 bpm and 1 patient had both SACT ≥ 140 msec and chronotropic response to atropine ≤90 bpm. In the Kaplan‐Meier analysis (primary analysis), time‐to‐event was significantly longer for the pacemaker‐ implanted patients (*P* < .001, Figure 2). From the 6 of 80 ABP patients with recurrent syncope on follow‐up, tilt‐table testing (TTT) was performed in 4 of them, being positive in 2. In two other patients, device interrogation revealed runs of NSVT leading to further ECG exploration with short term (24‐hour Holter monitoring) and signal‐averaged electrocardiography off pacing (during pacing at low VVI rate of 30 bpm in order to reveal the underlying rhythm and avoid the ABP noise producing artifacts). In one of these two paced patients a negative ventricular stimulation study followed.

**FIGURE 3:**
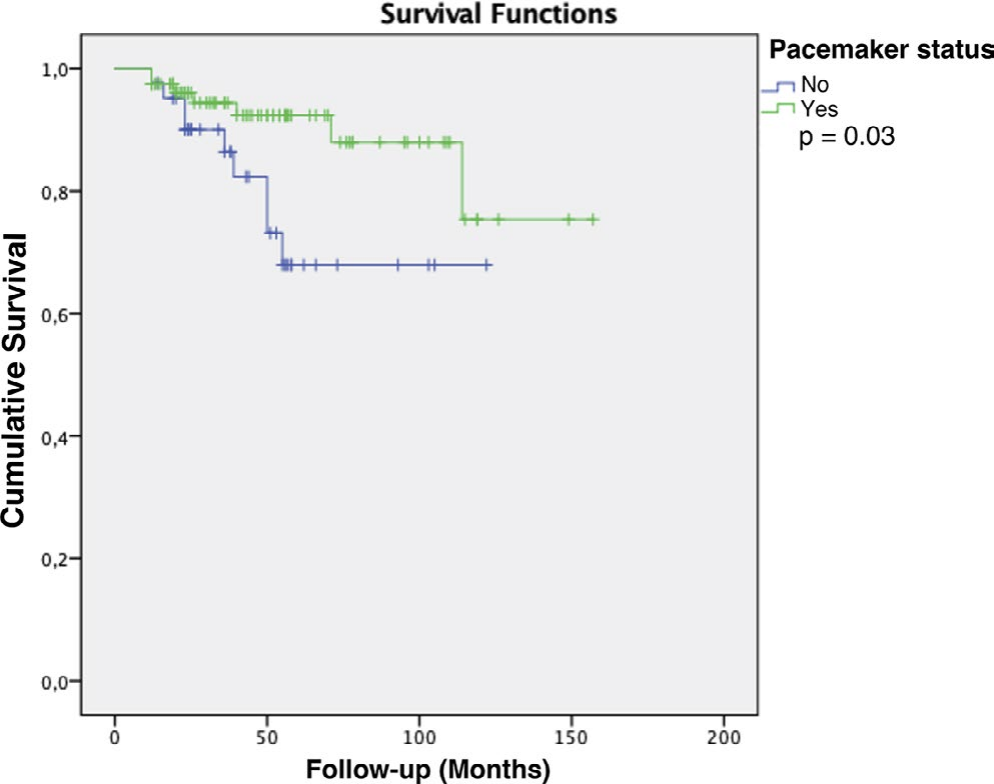
Kaplan‐Meier curves for death from any cause according to permanent pacing status

Sixteen patients died during follow‐up; cardiac causes were ascertained in 10. The estimated all‐cause mortality was 8.8% in the pacemaker group as compared to 21.4% in the no pacemaker group (*P* = .049). In the Kaplan‐Meier analysis, time‐to‐event was significantly longer for the pacemaker‐implanted patients (*P* = .030, Figure 3). The proportion of cardiac deaths was not different between paced and nonpaced patients (5% vs 14.3%, respectively; *P* = .079).

In the multivariable analysis, permanent pacing remained associated with syncope‐free survival in the predefined Cox regression model including age, ejection fraction, and organic heart disease: multivariable odds ratio 0.17 (95% confidence interval 0.06‐0.50). Pacemaker status was the only independent predictor of the primary event, associated with 83% decrease in the risk of recurrent syncope (Table 3). Adding 1st AVB, LBBB and Bifascicular block before EPS to the variables of the model did not change the result in any significant way.

**TABLE 3:** Cox regression model for recurrent syncope

Variables	Univariate			Multivariate^a^		
	OR	95% CI	*P*‐value	OR	95% CI	*P*‐value
Age	0.99	0.97, 1.01	0.477	1.01	0.99, 1.03	0.461
Organic heart disease	0.60	0.19, 1.86	0.375	0.39	0.04, 3.70	0.414
Ejection fraction	0.99	0.95, 1.05	0.922	0.98	0.93, 1.03	0.357
Pacemaker status	0.17	0.06, 0.48	0.001	0.17	0.06, 0.50	0.001

Abbreviations: OR, Odds Ratio; CI, Confidence intervals.

^a^ With backward elimination according to likelihood ratio criteria.

## 4 DISCUSSION

In this analysis of “real‐world” data in patients with mild degrees of SB, with a history of unexplained syncope, a set of positivity criteria for EPS evidence of SND identifies a subset of patients who will definitely benefit from permanent pacing, in terms of both preventing new syncopal episodes and reducing total mortality. A rather significant proportion of these SB/SND patients had an associated AVNCD based on the presence of both noninvasive and invasive abnormal electrophysiological parameters.

None of our syncope patients had severe SB or long enough pauses on the noninvasive electrocardiographic screening, to justify direct permanent antibradycardia pacing. Furthermore, the documentation of SND on EPS was supported by additional to those referred in the guidelines criteria of positivity.^4^, ^7^

Thus, even mild degrees of SB may be associated with EPS evidence of SND among recurrent syncope patients in whom atrioventricular pacing may suppress neurological symptoms during follow‐up. After a noninvasive ECG examination, a thorough EPS should follow in a combined EP guided risk stratification approach in such symptomatic SB patients. Within the advantages of such an approach are apart from the obvious clinical benefits, the avoidance of the need to resort to an implantable loop recorder policy which is associated with increased costs and the recurrent risk of injury secondary to syncope.^8^, ^9^, ^10^

In our study protocol we used quite “strict” criteria of abnormal electrophysiology test findings.^5^, ^11^, ^12^, ^13^, ^14^, ^15^ On the contrary, in the guidelines the sinus node function assessment was limited to the sinus node recovery time, ignoring other parameters such as the SACT and the chronotropic response to atropine.^4^ In agreement to our study results, the detection of SND on EPS is significantly improved when these parameters are examined in combination.^11^, ^13^ However, we have to admit the not uncommon coexistence of such mixed syncope reactions among SND patients undergoing TTT during the unexplained syncope baseline work‐up. Indeed, in two of four ABP SND patients with recurrent syncope on follow‐up, a neurocardiogenic mechanism was revealed in the subsequent TTT.^16^ Permanent pacing in these patients improves symptoms and frequently prevents recurrent syncope. Thus, it appears that a significant proportion of unexplained syncope patients with even borderline SB to be improved clinically with permanent pacing. Such a therapeutic approach may limit the high recurrence syncope rate reported in these patients undergoing electrophysiology test.^17^ In addition to this, a negative EPS was associated with a good long‐term prognosis even in the presence of LV dysfunction in patients with unexplained syncope.^18^ Although the role of EPS for the assessment of SND presence among transient SB patients has been questioned in the past,^19^ our data support its use in a rather significant proportion of unexplained syncope patients presenting with borderline degrees of SB.

Our study was a retrospective observational study with a nonimplanted self‐control group. Similar beneficial effects of pacing among vasovagal syncope patients have been observed in three small randomized controlled trials.^20^, ^21^, ^22^ Again, treatment in all three studies was not blinded, so that patients and their physicians knew whether the patient had received a pacemaker or not. On the contrary, two subsequent double‐blind randomized trials, one performed by the same group, suggested that pacing therapy did not reduce the risk of recurrent vasovagal syncope.^23^, ^24^ Whether the same principle holds for our SND/AVNCD patient group detected by strict EPS grounded criteria and not by the rather loose tilt table testing criteria of positivity for the diagnosis of neurocardiogenic syncope patient group is currently unknown.

It has been suggested that even symptomatic bradycardia patients have a benign long‐term prognosis as far as survival, regardless of initiation of permanent ABP.^25^ Interestingly, when looking at mortality, a lower proportion was seen in our SB patients receiving a pacemaker. Beyond the prevention of marked bradycardia or asystole, other significant effects of pacing may have contributed to the observed results. The associated increased incidence of a coexisting AVNCD among our SB/SND patients might have been an additional risk factor for bradycardia‐related cardiac mortality. A mortality reduction for pacing in sick sinus syndrome has never been shown previously, despite much larger multicenter studies.^26^ Thus, the results of our study in the mortality rate might have been influenced by the coexisting AVNCD. Indeed, a nonstatistical significant trend for an increased cardiac mortality was observed among our patients not receiving a pacemaker. Furthermore, we cannot exclude a beneficial effect of pacing on supraventricular tachyarrhythmia events occurrence known to be associated with SND and an increased mortality rate.^8^, ^17^, ^27^, ^28^

This is an observational, retrospective study. The main limitation is the nonrandomized nature of the permanent pacemaker implantation, based on physician judgement, introducing a potential for bias. Furthermore, self‐reporting of syncope episodes recall introduces bias (eg, such as misreporting because of respondent memory lapses, or miss‐estimation of the number of the episodes) to the number of syncope data. Baseline characteristics were largely similar between the two groups, but those with permanent pacemaker were somewhat older with a higher incidence of 1st AVB, LBBB and Bifascicular block. This may suggest that the pacemaker group was a higher risk group prior to treatment. In any case, multivariate analysis did not appear to alter the results.

In this analysis of “real‐world” data among patients with mild degrees of SB and a history of unexplained syncope, a set of positivity criteria for the presence of EPS defined SND after differentiating reflex syncope, identifies a subset of patients who will benefit from permanent pacing. A randomized control study of a combined EPS inclusive guided approach is needed in order to better define the best strategy of treating such patients, namely with EPS guidance or an implantable loop recorder documentation policy.

## DISCLOSURE

The authors declare no conflict of interest for this article.

## Data Availability

Data available on request.
